# *Mycobacterium chelonae* associated with tumor-like skin and oral masses in farmed Russian sturgeons (*Acipenser gueldenstaedtii*)

**DOI:** 10.1186/1746-6148-10-18

**Published:** 2014-01-14

**Authors:** Elisabetta Antuofermo, Antonio Pais, Sara Nuvoli, Udo Hetzel, Giovanni P Burrai, Stefano Rocca, Monica Caffara, Ilaria Giorgi, Claudio Pedron, Marino Prearo

**Affiliations:** 1 Department of Veterinary Medicine, University of Sassari, Via Vienna 2, 07100 Sassari, Italy; 2 Section of Animal Science, Department of Agriculture, University of Sassari, Via Enrico de Nicola 1, 07100 Sassari, Italy; 3 Department of Veterinary Bioscience, Faculty of Veterinary Medicine, University of Helsinki, Agnes Sjöbergin katu 2, FI-00014 Helsinki, Finland; 4 Department of Veterinary Medical Sciences, Alma Mater Studiorum, University of Bologna, Via Tolara di Sopra 50, 40064 Ozzano Emilia (BO), Italy; 5 State Veterinary Institute of Piedmont, Liguria and Aosta Valley, Fish Disease Laboratory, Via Bologna 148, 10154 Torino, Italy; 6 DVM, 20090 Settala, Milano, Italy

**Keywords:** *Acipenser gueldenstaedtii*, Aquaculture, *Calcinosis circumscripta*, Fish pathology, Mycobacteria

## Abstract

**Background:**

Non-tuberculous mycobacteria responsible for piscine mycobacteriosis usually produce visceral granulomas in both freshwater and marine species. In this study, the first occurrence of *Mycobacterium chelonae* associated with tumor-like lesions in the Russian sturgeon (*Acipenser gueldenstaedtii*) is reported. Fifteen sturgeons from an Italian fish farm showing skin and oral cauliflower-like masses were investigated by histopathology, bacterial culture and molecular analyses.

**Results:**

A total of 20 masses different in size located in the mouth and in pectoral and caudal fins (characterized by abundant calcium deposits and by mild to moderate granulomatous inflammation) were observed with a significant different degree of histological severity. All internal organs of the fish were negative for mycobacteria, Ziehl-Neelsen was positive in only one of the oral masses, whereas bacterial and PCR analyses detected the presence of *M. chelonae* for almost all the skin and oral masses. Based on these results, a calcinosis of dystrophic origin associated with a chronic granulomatous inflammation was considered as a primary diagnosis consequent to tissue injury in areas susceptible to trauma.

**Conclusions:**

We hypothesized that the occurrence of *M. chelonae* in farmed sturgeons was only a secondary event related to its presence in a stressful rearing environment and subsequent to a dystrophic calcinosis occurred in previously damaged tissues.

## Background

The Russian sturgeon, *Acipenser gueldenstaedtii* Brandt & Ratzeburg, 1833, is an ancient fish native to Black Sea, Sea of Azov and Caspian Sea entering all the main rivers that empty into them [1].This species has both anadromous and freshwater populations: at sea, it occurs in shallow coastal and estuarine zones, while in freshwaters it inhabits the deep parts of large rivers with moderate to swift current [2]. It was introduced throughout Europe and, due to loss of habitat caused by the construction of dams and to overfishing to collect its eggs, it is now considered critically endangered although it is a promising species for aquacultural purposes [3-6].

In recent decades, sturgeon farming for meat and caviar production has increased in Europe [7]. Nevertheless, the development of sturgeon aquaculture activities has been frequently accompanied by disease outbreaks, including those unknown prior to farming. Unfortunately, research on the major sturgeons diseases had not received much attention in the past and, therefore, the knowledge on their prevalence, distribution, origin and ecological significance is still fairly limited [8]. Although sturgeons are considered relatively resilient to diseases, several bacterial, viral and parasitic diseases have been reported worldwide [8], including Columnaris disease [9-11], motile *Aeromonas* septicemia [12-14], yersiniosis [15], pseudomonosis [16], epitheliocystis [17], white sturgeon adenovirus [18,19], herpesvirus [20,21] and iridovirus [22-25].

To date, no reports of skin and/or oral tumor-like masses in sturgeons associated with mycobacterial infections are documented. Only few papers described a group of Siberian sturgeons (*Acipenser baeri*) imported in Italy from France showing visceral granulomas [26], and caviar fishes from Iran exhibiting gill lesions, both caused by *Mycobacterium marinum*[27].

The non-tuberculous mycobacteria (NTM), also known as environmental mycobacteria (EM), atypical mycobacteria (AM) and mycobacteria other than tuberculosis (MOTT), include those *Mycobacterium* species which are not members of the *Mycobacterium tuberculosis* complex (and *M. leprae*) [28]. NTM have a worldwide distribution as saprophytes in soil and in treated/untreated waters, where they can remain viable for over 2 years. NTM include many species of acid-fast bacteria, some of which are capable of causing chronic progressive diseases in mammals, birds, reptiles and fish. Thus, infected fish may be considered as primary reservoir for these pathogens and infection is probably acquired from NTM present in the environment by ingesting contaminated food or water containing organic matter, or through gill or skin lesions caused by trauma and parasites [29]. Mycobacteria may cause cutaneous lesions or spread into internal organs through the circulatory or lymphatic systems. Transovarian transmission has also been documented in Siamese fighting fish (*Betta splendens*) [30]. All fish species should be considered susceptible to mycobacteriosis, with a higher prevalence in farmed animals and significant losses frequently observed in aquacultural activities [31]. However, mycobacteriosis infections and mass mortalities in wild fish is not easy to recognized but see [32], because infected fish die and are easily predated or scavenged.

Disease outbreaks in farmed fish can be related to several management features, such as the quality and quantity of food and water supplied, and the rearing density. As a result, inadequate management could cause abnormal stress associated with a decrease of the normal resistance of the host [30].

Mycobacteriosis in fish is currently attributed to a wide array of species belonging to the *Mycobacterium* genus, but historically *M. chelonae*, *M. fortuitum*, and *M. marinum*[31,33] are the most reported species. Furthermore, *M. marinum* is the most common in a wide range of saltwater fish [34] and *M. chelonae* (*M. salmoniphilum*) has been identified in different coldwater fish, especially in salmonids [35,36].

In general, piscine mycobacteriosis is a systemic subacute to chronic progressive disease causing grey-whitish nodules (granulomas) and enlargement of various organs (mainly spleen, liver and kidney) [37-39]. Clinical signs are often lacking and hardly detectable until advanced stages, in which cachexia, lordosis, pale gills and skin ulcerative lesions can be seen in some fish species [30]. Skin granuloma caused by NTM infection was not reported in any fish species, except for Atlantic salmon farmed in British Columbia [35].

However, to our knowledge, no skin or oral granulomatous inflammation with excessive mineralization associated with mycobacteria has ever been observed in sturgeons. The aim of this study, therefore, was to describe in detail the first occurrence of tumor-like lesions associated with *Mycobacterium chelonae* in the Russian sturgeon, *Acipenser gueldenstaedtii*.

## Methods

### Fish sampling

The study was carried out in a fish farm located in Northern Italy (Ticino basin, Province of Pavia), where several species of sturgeons are reared in raceways system at different density depending on the size of fish. Several juveniles belonging to the same aquaculture production batch (<2 years old) of *Acipenser gueldenstaedtii* showed skin and oral masses. Fifteen live sturgeons sampled in July 2010 were euthanized by immersion in a lethal dose of tricaine methanesulphonate (MS-222, Sigma-Aldrich), and a complete necropsy of each subject was performed. The sampling was performed in accordance with the Italian Legislative Decree n. 116/1992, in application of the EU Directive 86/609/EEC. For this study, ethical approval from an institutional ethical committee was not required because all the sturgeons examined were collected as a part of a plan of surveillance and sanitary controls on fish health.

The lesions were localized at 3 different levels: oral (lips and cheeks), pectoral, and caudal fins and were categorized according to their size in 3 groups: small (from 0.2 to <0.5 cm), medium (from 0.5 to 2 cm) and large (more than 2 cm). Furthermore, voluminous skin masses (10 to 20 cm in diameter) were also observed in older sturgeons (5-6 years old). Due to their high commercial value, these fish were not killed and consequentially their lesions were not evaluated in detail. Furthermore, 10 healthy sturgeons were sampled from the same raceway as negative controls.

### Cytopathology and histopathology

Fine needle aspiration cytology of oral and skin masses were performed and smears were stained with May Grünwald Giemsa (MGG) and Ziehl-Neelsen (ZN). Samples of all lesions were formalin fixed, paraffin embedded and submitted to histopathological evaluation. Slides were stained with Hematoxylin and Eosin (HE), Ziehl-Neelsen (ZN), Von Kossa and Masson’s Trichrome stain and observed at light microscopy level. Based on the inflammatory response, mineralization, necrosis and fibroplasia evaluated, the histopathological lesions were scored in 3 levels of severity (mild, moderate, and severe) by two different pathologists. Photomicrographs were acquired with a Nikon Digital Sight DS-U1 camera mounted on a Nikon 80-i microscope.

### Bacteriological and molecular diagnosis

Skin and oral lesions as well as visceral organs (spleen, kidney and liver) were collected from each fish. Tissue samples were homogenized, decontaminated using 1.5% cetylpyridinium chloride (Sigma-Aldrich) for 60 min. and centrifuged at 3000 rpm for 20 min. Tissue homogenates (10 μl) were spread onto a glass slide, stained with ZN, and other 10 μl cultured on Löwenstein-Jensen media tubes incubated at 25±1°C and 37±1°C. Possible mycobacterial growth was assessed every day for a period of 1 month and the isolated colonies were evaluated by growth and biochemical test [40]. Test tubes were maintained for 2-3 months before being considered negative.

Total DNA was extracted from the colony by QIAamp DNA Mini Kit (Qiagen). A fragment of ~439 bp of the 65-kDa heat shock protein gene (*hsp*65) was amplified by two specific primers: Tb11 5’-ACC AAC GAT GGT GTG TCC AT-3’ and Tb12 5’-CTT GTC GAA CCG CAT ACC CT-3’ [41]. The PCR was carried out in 50 μl reactions containing 10X PCR buffer (Invitrogen), 200 μM dNTPs (Invitrogen), 0.3 μM of each primer, 1.5 mM MgCl_2_ and 2.5 U Platinum Taq DNA Polymerase (Invitrogen). A Tpersonal (Biometra) thermocycler was used for the amplification with the following parameters: 45 cycles of 1 min at 94°C, 1 min at 55°C and 1 min at 72°C, preceded by 5 min denaturation step at 95°C and finished with an extended elongation step at 72°C for 10 min. The PCR products were electrophoresed on a 1% agarose gel (Sigma) stained with SYBR Safe DNA Gel Stain in 0.5X TBE (Molecular Probes-Invitrogen). The PCR product were digested with the restriction enzyme *Bst*EII and *Hae*III (MBI Fermentas) following the manufacturer’s protocols [41]. Restriction fragments were separated by 3% agarose gel (Sigma) stained with SYBR Safe DNA Gel Stain in 0.5X TBE and the band size were determined by the computer program Quantity One 4.4.1 (Biorad) with 100 bp molecular size standard (XIV Roche). In order to avoid analysis mistake, the digested strains were first separated on the basis of the phenotypic pattern and then electrophoresed together with the specific reference strain (DMSZ 43804/ATCC 35752, Leibniz-Institut, German Collection of Microorganisms and Cell Cultures, Braunschweig, Germany).

### Statistical method

Macroscopical and microscopical features of the lesions were analyzed using Stata 11.2 software (StataCorp LP). Differences between the size of the lesions and their degree of histological severity in the mouth and in pectoral and caudal fins were compared using the non-parametric Kruskal-Wallis test with Dunn’s *post-hoc* comparison [42]. Furthermore, categorical and ordinal variables were compared using the Spearman rho (*ρ*) rank correlation coefficient. A value of *ρ* approximately equal to 1 indicates a good correlation, a value near 0 indicates a poor correlation, and a negative value indicates an inverse correlation. A *P*-value <0.05 was considered significant.

## Results

### Macroscopical findings

**Figure 1:**
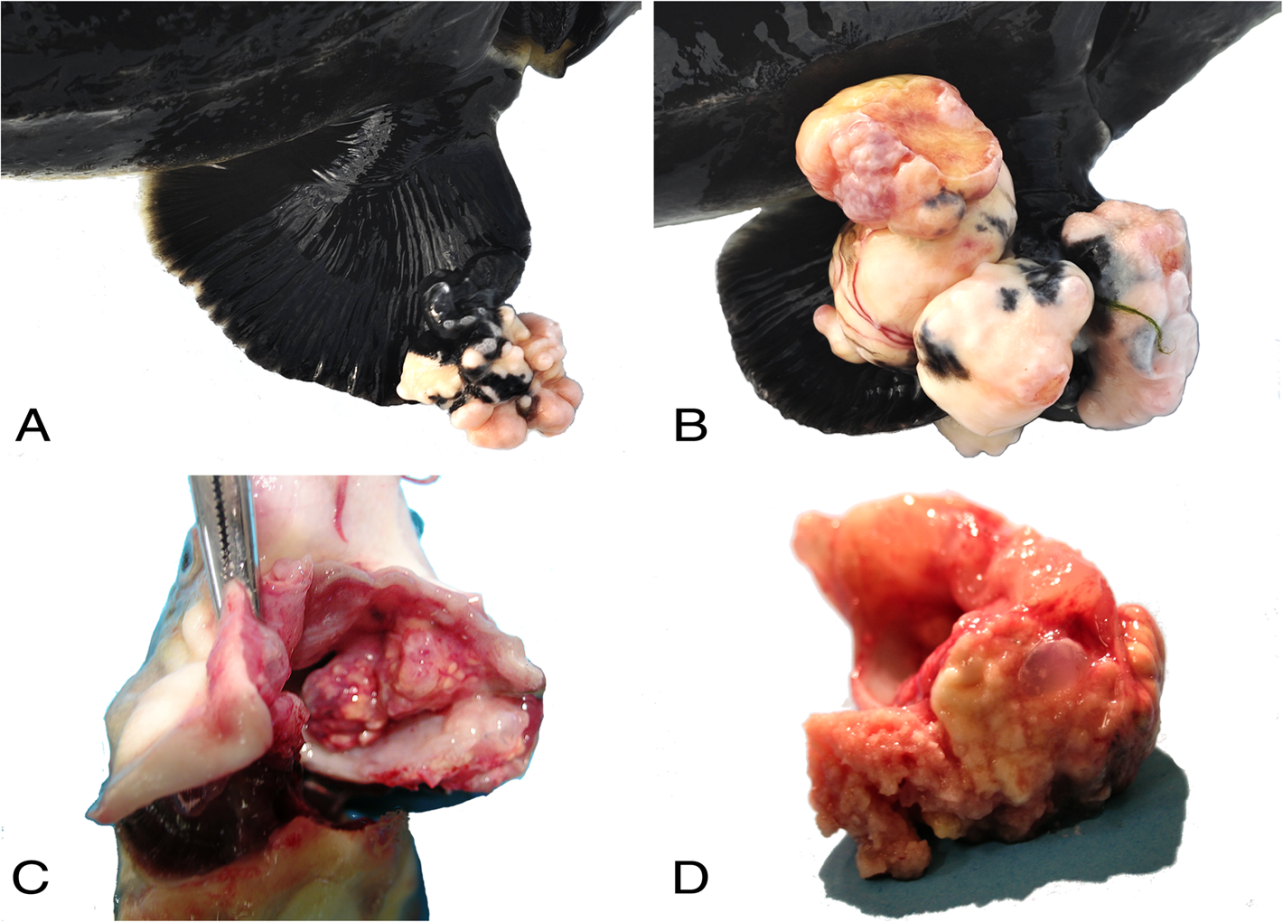
**Macroscopic findings. (A)** Pectoral fin: multiple cauliflower-like masses of medium size**. (B)** Pectoral fin: multiple large-sized ulcerated masses. **(C)** Oral cavity: large-sized lesions infiltrating the submucosa. **(D)** Detail of the Figure 1C: yellowish pasty component of the lesions.

At postmortem examination, a total of 20 skin and oral cavity masses were found in the 15 sturgeons examined. The 73% of the fish exhibited single lesions, mostly located in the mouth (82%), although 27% of the sturgeons showed multiple skin and oral masses. Total skin lesions accounted for 35%, and were located in both pectoral (43%) and caudal fins (57%). These lesions were mostly medium-sized (Figure 1A), except for one large (Figure 1B) found in case No. 7 and another small one (case No. 1) located in the pectoral and caudal fins, respectively (Table 1). Grossly, all the skin masses were firm, whitish in color and showed a cauliflower-like exophytic growth (Figure 1A and B). Oral cavity masses (Figure 1C) accounted for 65% and were significantly larger (>2 cm) compared to those on the skin (Kruskal-Wallis H = 12.504, *P* < 0.01; Dunn’s *post-hoc* test, mouth *vs* caudal fin Q = 2.622, *P* < 0.05). Oral masses were mostly infiltrative within the submucosa and, on cut section, were composed of a yellowish gritty material (Figure 1D). No one of the sturgeons examined showed visceral lesions.

**Table 1:** Location and size of oral and skin masses in affected sturgeons

**Case #**	**Lesion #**	**Location**	**Lesion size**	**Histological severity**	**ZN**	**Culture**
1	1	Caudal fin	Small	Mild	Negative	Negative
2	1	Mouth	Medium	Moderate	Negative	Negative
3	1	Mouth	Large	Severe	Positive	Positive
4^a^	1	Mouth	Large	Severe	Negative	Positive
	2	Caudal fin	Medium	Moderate	Negative	Negative
	3	Pectoral fin	Medium	Severe	Negative	Positive
5	1	Pectoral fin	Medium	Severe	Negative	Negative
6	1	Mouth	Large	Severe	Negative	Positive
7^a^	1	Mouth	Large	Severe	Negative	Positive
	2	Pectoral fin	Large	Moderate	Negative	Negative
8	1	Mouth	Large	Severe	Negative	Positive
9	1	Mouth	Large	Severe	Negative	Positive
10^a^	1	Mouth	Large	Severe	Negative	Positive
	2	Caudal fin	Medium	Moderate	Negative	Negative
11	1	Mouth	Large	Severe	Negative	Negative
12	1	Mouth	Large	Severe	Negative	Negative
13^a^	1	Mouth	Large	Severe	Negative	Negative
	2	Pectoral fin	Medium	Moderate	Negative	Negative
14	1	Mouth	Large	Severe	Negative	Negative
15	1	Mouth	Large	Severe	Negative	Negative

^a^Sturgeons with multiple lesions.

### Cytopathology and histopathology

Grossly, unstained smears showed a chalky, white material. Cytologically, the aspirate exhibited a large amount of amorphous granular, dark-gray to bluish material admixed with glassy retractile fragments, suggestive of calcium salt deposit, uniformly dispersed in the background. Cellular components were represented by a moderate to severe mixed inflammatory reaction (macrophages, lymphocytes and multinucleated giant cells) and numerous reactive spindloid, fibroblast-like cells. There was no evidence of bacteria or other infectious agents and ZN was negative for acid-fast bacilli.

**Figure 2:**
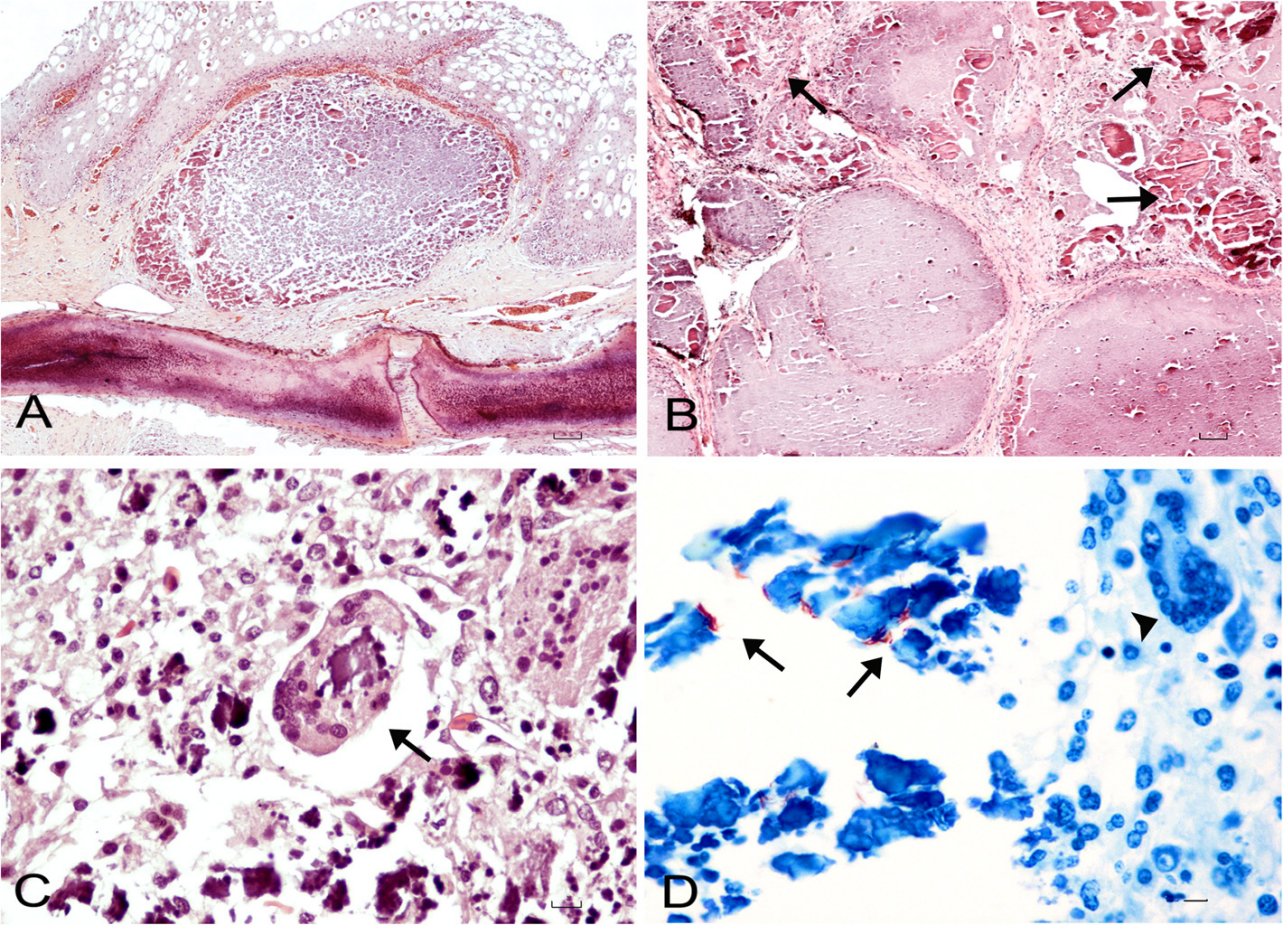
**Histopathology. (A)** Caudal fin: well demarcated nodular subcutaneous lesion covered by epidermis (HE stain, bar = 100 μm). **(B)** Oral mass: poorly demarcated multinodular lesion infiltrating the submucosa with several foci of mineralization (arrows) scattered throughout the masses (HE stain, bar = 100 μm). **(C)** High-power magnification of Figure 2B: severe mononuclear inflammatory infiltrate with a multinucleated giant cell (arrow) containing phagocytized calcium (HE stain, bar = 100 μm). **(D)** Oral mass: few red rod-shaped extracellular acid-fast bacteria (arrows) and occasional giant cell not containing phagocytized mycobacteria (arrowhead) (ZN stain, bar = 100 μm).

On histopathology, skin masses showed generally a multinodular aspect, with an infiltrative growth pattern into the dermis and subcutis, surrounded by abundant fibrous stroma. Only one caudal fin skin mass (case No. 1) exhibited a nodular pattern and was well encapsulated and demarcated (Figure 2A).

Skin lesions were mostly characterized by mild to moderate deposition of amorphous granular calcium material, positive to Von Kossa stain, and by mild to moderate inflammations composed mostly of macrophages, lymphocytes, and few giant cells. Mild to moderate fibroplasia was also evident with Masson’s Trichrome stain. Oral masses were characterized by a multinodular infiltrative pattern into the submucosa and were poorly demarcated and nonencapsulated (Figure 2B). Moderate to severe inflammatory reaction with a high number of macrophages, lymphocytes and numerous giant cells, phagocytizing calcium debris were found (Figure 2C). Excessive mineralization deposit associated with severe fibroplasia and necrosis was also observed. Red, rod-shaped extracellular acid-fast bacteria within mineralized deposits of calcium and necrotic debris were seen in one of the oral masses (case No. 3) examined (Figure 2D).

Fourteen out of twenty lesions (70%), mostly found in oral cavity (86%), exhibited severe histological changes, and showed a higher degree of severity than those located in the skin (Kruskal-Wallis H = 11.020, *P* < 0.01; Dunn’s *post-hoc* test, mouth *vs* caudal fin Q = 2.578, *P* < 0.05). Spearman correlation analysis found a significant positive correlation only between the size of the lesions and their histopathological severity (ρ = 0.7028, *P* < 0.001).

### Bacteriological and molecular results

None of the smears obtained from the masses sampled were positive to ZN stain. Colonies growth from cultured oral and skin masses, were clearly visible in Löwenstein-Jensen media tubes showing acid-fast bacilli at ZN stain (both tubes incubated at 25°C and 37°C were positive). These colonies were phenotypically and biochemically identified as *Mycobacterium chelonae.* All the internal organs cultured were negative for mycobacteria. Furthermore, no colony growth of mycobacteria were observed from all negative controls.

**Figure 3:**
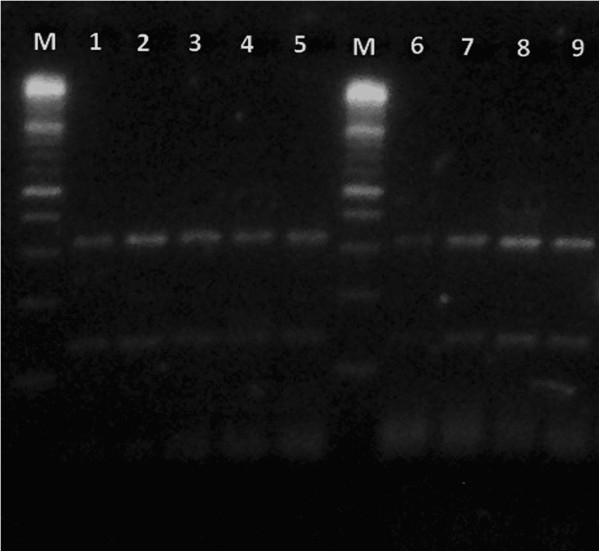
**Molecular analysis.***BSte*II enzyme. M = 100 bp molecular weight standard; lanes 1-8 = samples; lane 9 = reference strain of *Mycobacterium chelonae* (DMSZ 43804/ATCC 35752).

**Figure 4:**
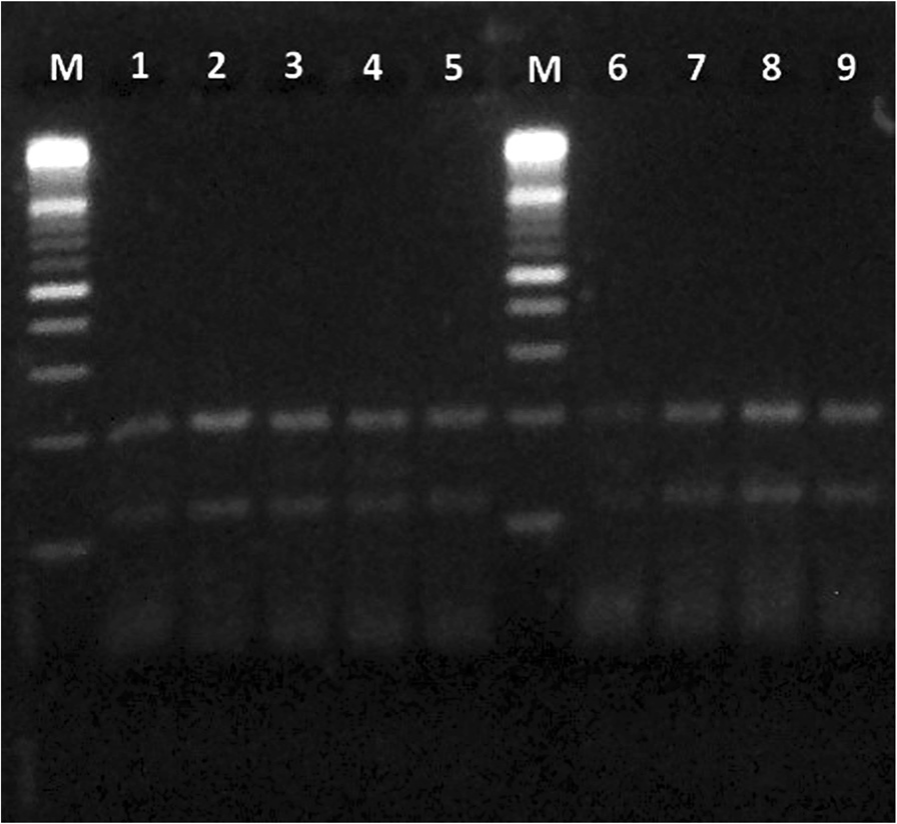
**Molecular analysis.***Hae*III enzyme. M = 100 bp molecular weight standard; lanes 1-8 = samples; lane 9 = reference strain of *Mycobacterium chelonae* (DMSZ 43804/ATCC 35752).

All the strains subjected to PCR-RFLP analysis showed a restriction pattern identical to the one of *M. chelonae* (Figures 3 and 4), confirming the results obtained by phenotypic and biochemical identification.

## Discussion

In the present study, the occurrence of *Mycobacterium chelonae* associated with tumor-like masses with excessive calcium deposition was described for the first time in farmed sturgeons. In general, the presence of this mycobacterium species and its association with gross lesions is considered quite rare in fish, with few reports in the literature. These records described *M. chelonae* mostly associated with granuloma-like visceral nodules in farmed salmon and turbot [35,43,44], in wild perch [45], and also in ornamental fish [39]. Furthermore, *M. chelonae* was evidenced in several other wild and domestic animals but mostly associated with ulcerative skin lesions [46] and references therein [47].

In all the sturgeons examined in this study, the lesions were different in size and larger masses were mostly observed in the oral cavity, most of which showing a severe histological pattern significantly correlated with their size and with an infiltrative activity. Moreover, all the skin and mouth lesions were characterized by different severity levels of chronic granulomatous inflammation. To the best of our knowledge, granulomatous infiltrates associated with few bacteria, isolated and confirmed by PCR as *M. chelonae,* were described in salmon [35]. It is worth noting that none of the lesions we observed showed typical granulomas, although these structures are quite common in fish organs with mycobacteriosis [31] and references therein. In particular, typical granulomas are characterized by peripheral fibrosis and several layers of epithelioid cells surrounding necrotic areas containing a large amount of acid-fast bacilli [33,48]. Conversely, we observed scant ZN positive bacilli intermingled with abundant mineralization, tissue necrosis and fibroplasia. Strong positivity to Von Kossa stain of mineralized material confirmed that calcium salt deposition was diffusely present within nodular and multinodular masses and increased from smaller to larger lesions. In general, mineralization is rarely encountered in piscine mycobacteriosis [33] and references therein. Nevertheless, ectopic mineralization in soft tissues has been reported for many other fish species [49] and references therein, although different names to describe similar lesions have been used, leading to confusion in terminology. In particular, an ectopic mineralization characterized by tumor-like nodules in subcutaneous tissues containing calcium deposition is defined as *Calcinosis circumscripta* (CC) [49] and references therein.

Thus, due to the macro and microscopic aspect of the masses we examined (that were all characterized by single or multiple nodules containing abundant calcium deposits), their distribution (single or multiple) and localization (skin and oral mucosa), CC syndrome was considered as a possible diagnosis [49] and references therein. This syndrome is a disorder affecting humans and several animal species (but never described in sturgeons before) and is classified in 3 different forms: metastatic, idiopathic and dystrophic calcinosis [49] and references therein.

Aetiopathogeneses for calcinosis include trauma, foreign body reaction, neoplasms, connective tissue diseases, abscesses, granulomas, inherited disorders and infections [50]. Generally, the ectopic calcified masses consist of hydroxyapatite and amorphous calcium phosphate [51]. It has been hypothesized that the denatured proteins of necrotic cells can serve as a core for ectopic calcification, and that the following degeneration of the collagen fibers and subcutaneous fat tissue can induce the calcification process [51]. In addition, inflammation and fibrosis may be frequently observed as a reaction of calcified deposits (e.g. foreign body reaction).

From a clinical point of view, nodules of calcium deposits can cause skin ulcerations occurring in areas of recurrent microtraumas. Thus, most probably, the calcinosis we observed in the sturgeon masses was of dystrophic origin, because tissue injures especially occurred over body prominences (e.g. pectoral and caudal fins), or in areas susceptible to trauma (e.g. mouth). Probably, therefore, sturgeons affected by these masses can be assumed to have feeding problems, leading to starvation. Furthermore, the fact that a number of sturgeons exhibited mouth and skin lesions could be related to farming conditions and, in particular, to stressful breeding density. As a consequence, the mycobacteria observed inside the sturgeon masses could have been present in the rearing environment (i.e. the water) and only after traumatic events infected the fish lesions [31]. Based on this hypothesis, we can suppose that the granulomatous inflammation process could be related to the presence of a foreign body reaction (i.e. the calcium) [49], and the occurrence of *M. chelonae* associated with tumor-like skin and oral lesions was only a secondary event. In fact, this mycobacterium is a fast growing, opportunistic bacillus ubiquitous in nature, exceptionally pathogenic and mainly associated to chronic granulomatous infections in immunocompetent and immunosuppressed subjects [31]. The histological pattern observed in almost all the sturgeon lesions (19 out of 20; 95%) seems to confirm this hypothesis, because no acid-fast bacilli were noticed in the macrophages cytoplasm, in epithelioid cells, nor in multinucleated giant cells which contained almost only calcium. Only in one of the oral lesion examined, we found a few extracellular acid-fast bacteria intermixed calcium deposits. However, a number of studies demonstrated that the sensitivity of some piscine mycobacteria to ZN staining could be referred to their physiological state [52], with acid-fast bacilli easily visible in mature granulomas, but difficult to observe in early lesions [31] and references therein.

For all the above reasons, we are reasonably doubtful in making a definitive diagnosis of mycobacteriosis for the *Acipenser gueldenstaedtii* specimens examined, although the presence of *M. chelonae* was detected by both tissue cultures and PCR-RFLP analysis. Nevertheless, we may not completely rule out that the lesions we observed could be primarily caused by *M. chelonae*, and a diagnosis of an unusual mycobacteriosis in sturgeons could be made. Thus, the excessive mineralization found in the lesions could be considered the result of a long standing granulomatous inflammation caused by *M. chelonae*. In fact, it could be supposed that sturgeons show an aberrant response to mycobacterial organisms in subcutaneous or mucosal localization leading to a pathological accumulation of calcium in inflammation reaction. Sturgeons, ancient fishes with a mostly cartilaginous endoskeleton, have smaller calcium requirements for calcification than other fish species and deposit significant amounts of calcium in their exoskeleton (i.e. the ganoid scale system and cranium), a feature that is unique to this group of vertebrates [53]. However, although many research efforts have been made in explaining the extracellular matrix calcification (ECM), the pathogenic mechanism causing ectopic deposition of calcium in sturgeon soft tissues remains still unknown [54]. Actually, among a number of molecules involved in the pathways of ectopic mineralization, vitamin K-dependent proteins (VKD) are known to play a important role in this pathogenesis [54].

In particular, a new type of VKD, called Gla-Rich Protein (GRP), which importance in normal and pathological deposition of calcium in tissues is still not completely understood, has been recently isolated in the Adriatic sturgeon, *Acipenser naccarii*[55].

To our knowledge, this unusual occurrence of *M. chelonae* associated with tumor-like masses with excessive mineralization was never described before in sturgeons and, in general, in fish. It is worth noting that the probability to find mycobacterial infections is higher in farmed fishes than in wild ones, as already pointed out in a recent study for several freshwater species [56]. *M. chelonae* is considered a human pathogen associated with infection of wound in immunocompromised person. However, because the strains of *M. chelonae* grow at different temperature (28.5°C or 37°C), further research is thus needed to definitely clarify the relation between the potential risk of the presence of *M. chelonae* in farmed sturgeons also for people working in aquaculture activities [33,57].

## Conclusions

The presence of *Mycobacterium chelonae* in skin and oral masses of the farmed specimens of *Acipenser gueldenstaedtii* reported in this study was considered as a secondary event subsequent to a dystrophic calcinosis occurred in previously damaged tissues. In fact, the absence of typical granulomas in all organs of the fish examined lead us to exclude a primary role of the piscine mycobacteriosis. Finally, experimental *in vivo* studies will be helpful to clarify the role of *M. chelonae* as a potential pathogen (or opportunistic agent) causing skin and subcutaneous tumor-like masses with calcinosis in sturgeon.

## Competing interests

The authors declare that they have no competing interests.

## Authors’ contributions

EA: designed, interpreted macro and microscopic findings and draft the manuscript; AP: performed the statistical analysis and draft the manuscript; SN: performed and interpreted the cytopathology and histopathology; UH: have been involved in the interpretation of histopathological results and critically revised the manuscript; GPB, SR: performed, interpreted the statistical analysis and prepared figures; IG, MC: performed and interpreted the bacteriological and the molecular analysis; CP: have been involved in the conception of the study and performed fish sampling; MP: designed, coordinated the study and revised the manuscript for important intellectual content. All authors read and approved the final manuscript.
